# The social significance of golf in Finland in the year 2021 based on SROI analysis

**DOI:** 10.3389/fspor.2026.1832817

**Published:** 2026-06-24

**Authors:** Julia Kettinen, Timo Ala-Vähälä, Andrew Murray, Charlie Foster, Nils Horn, William R. Taylor, Raija Laukkanen

**Affiliations:** 1 Laboratory for Movement Biomechanics, ETH Zürich, Zürich, Switzerland; 2 Balgrist Golf Institute, Balgrist University Hospital, Zürich, Switzerland; 3 Institute of Biomedicine, Sports and Exercise Medicine, School of Medicine, University of Eastern Finland, Kuopio, Finland; 4 Finnish Society of Sports Sciences Helsinki, Helsinki, Finland; 5 Sport/Northlight, University of Edinburgh, Edinburgh, United Kingdom; 6 European Tour Health & Performance Institute, European Tour Group, Surrey, United Kingdom; 7 Bristol Medical School, University of Bristol, Bristol, United Kingdom; 8 Faculty of Medicine, University of Oulu, Oulu, Finland

**Keywords:** adults, golf, health, physical activity, social impact, SROI, wellbeing

## Abstract

**Introduction:**

There is growing demand among policy makers to understand the broader social impact of different recreational activities towards supporting policy interventions and promoting societal benefits through active lifestyles. Golf is a growing global sport and economic contributor, particularly among middle-aged and older adults, whose participants have high and increasing healthcare costs.

**Methods:**

This study analysed the social return on investment (SROI) of golf in Finland in 2021 across two dimensions: (1) the financial flows generated by Finnish golf players’ spending across various economic sectors, and (2) the wellbeing and physical fitness promoted by golf participation and their broader effects on societal sectors such as healthcare services. A cross-sectional online survey was conducted in 2022 among members of the Finnish Golf Union and its affiliated golf clubs (*n* = 1,052) and a review of financial statements from ten golf courses. The SROI ratio of golf was calculated as total societal benefits from golf/total spending on golf.

**Results:**

Golf generated substantial societal benefits in Finland. Total societal benefit comprised player wellbeing and joy (€330M), measurable economic contributions (€220M), and societal savings and added revenues (€80M), totalling €630M. With total player spending of €330M, the SROI ratio was 1.9. Applying Finland's Leontief coefficient (1.7) for sports and recreation services increased economic impact to €370M and total benefits to €770M, raising the SROI ratio to 2.4.

**Conclusion:**

Golf participation generates substantial wellbeing and economic benefits at individual and societal levels, highlighting its potential as a health-promoting and economically significant recreational activity.

## Introduction

There is a globally growing demand and interest among policy makers and practitioners to understand and measure the social impact of different types of recreational activities to justify policy makers support for strategies that deliver societal benefits from active lifestyles (1, 2). Regular physical activity is one of the best non-pharmacological ways to improve health, and supports many health and social benefits such as disease prevention, prosocial behavior, and psychological and cognitive benefits (1, 3). Physical inactivity is a major modifiable risk factor for non-communicable diseases and mental health conditions (4), and it is associated with higher healthcare costs for populations (5). It is estimated that if current physical activity levels remain globally at the same level, 499 million new cases of preventable non-communicable diseases and mental health conditions would occur from 2020 to 2023, and that the total global cost of physical inactivity over this same period would reach approximately INT$520 billion (4). Particularly among middle-aged and older adults, physical activity interventions promote healthy behavior (6) and subsequent societal benefits. However, from a healthcare cost perspective, the prevention of physical inactivity–related non-communicable diseases may also lead to higher costs due to increased longevity (5), which underscores the importance of assessing the broader social significance of recreational activity and physical activity in supporting individuals’ functional ability during later years.

In addition to health benefits, there is a need for better understanding how specific types of recreational activity contribute to society. The social return on investment (SROI) methodology enables the quantification of wider socio-economic outcomes in a single monetary ratio, highlighting physical activities that can reduce the social and economic burden of non-communicable diseases and improve the wellbeing of the population (5). SROI assesses how physical activity and sport generate societal benefits by converting intangible outcomes into monetary equivalents (7). For example, Davies et al. (8) applied the SROI framework to 12 community sport and leisure facilities in Sheffield, including golf courses, and found that for every €1.15 invested, a social return of between €1.39 and €3.95 was generated. Nevertheless, there is currently no consensus on which social outcomes should be measured and how they should be evaluated (2). This is further supported by Stielke et al. (9), whose scoping review of 21 SROI studies on physical activity interventions found that only four were published in peer-reviewed journals, highlighting the need for more rigorous academic research in this field.

In Finland, previous research on the broader societal impacts of physical activity has examined both the direct and indirect expenses resulting from insufficient physical activity and high levels of sedentary behavior (10). The primary economic impacts considered direct healthcare and eldercare costs, as well as indirect costs and lost benefits, such as sickness-related absences, disability pensions, unemployment benefits, all-cause mortality, and losses in income tax revenue. The economic impacts of insufficient physical activity, including both direct and indirect expenses as well as lost benefits, amounted to approximately €3.2 billion per year, representing about 1.4% of Finland's gross national production in 2017. In a separate analysis, Kolu et al. further estimated the costs of sedentary behavior at approximately €1.5 billion. These figures underscore the potential value of understanding how recreational activities such as golf could contribute to reducing these substantial public health costs.

Golf is a growing global sport and economic contributor, especially among middle-aged and older adults, who represent the largest potential age group for reducing healthcare costs (6) and increasing economic activity through recreational spending. Golf participation is substantial worldwide, with over 100 million people playing the sport globally (11). The COVID-19 pandemic accelerated engagement in golf-related activities (11), performed on golf courses and driving ranges/practice areas, as these outdoor settings allowed for safe social distancing (12, 13). During pandemic lockdowns, when golf was deemed both acceptable and safe, individuals who had previously been only marginally engaged were reactivated and reconnected with the sport (11). This momentum of growing interest in golf has been maintained post-pandemic (11). This may indicate that the game continues to fulfill fundamental motivational factors individuals seek from recreational activities, such as social connection, enjoyment, improved psychological and physical condition, skill improvement, and mastery (14). Beyond traditional on-course golf participation, golf-related off-course activities, including technology-enabled driving ranges, golf simulators, and entertainment venues, have experienced significant growth in recent years (15).

From the perspective of supporting active lifestyles and preventing non-communicable diseases, golf offers a distinctive combination of moderate-intensity aerobic exercise, muscle engagement, cognitive stimulation, social interaction, mental health benefits, and outdoor activity in green spaces that reduces sedentary time (13, 16–20). This multifaceted nature of golf is particularly valuable for middle-aged and older adults. Previous studies focusing on Finnish golfers show that Finnish golf players meet WHO's physical activity recommendations across all age groups throughout the year, despite seasonal variation (21, 22). Promising results have also been reported from the “Golf for Health” intervention programme in Scotland, which targets physically inactive non-golfers, introducing golf as an accessible and socially engaging activity with potential long-term health and wellbeing benefits (23). Similar results have also been reported from the Golf in Society project, which aims to improve the lives of the ageing population with conditions such as dementia, Parkinson's disease, and depression by introducing them to the health and wellbeing benefits of golf and promoting social inclusion for participants and carers (24).

Golf has substantial economic impact globally. In the United States, the total economic impact of golf, including indirect and induced effects, reached €193 billion in 2022, of which €87 billion came from direct economic activity, representing an increase of approximately 20% since 2016 (15). In Europe, total economic impact was estimated at €15 billion in 2013 (25), with the European golf tourism market alone generating €6.8 billion in 2025 (26). Despite golf's economic impact, there is a notable gap in research examining the social return on investment (SROI) of golf. In the UK, the first national SROI-based analysis estimated that golf participation and volunteering generated €1.20 billion in social value in 2019, primarily through improved subjective wellbeing, community development, and health outcomes (27). A particularly compelling perspective for SROI analysis is that golf participants often cover the costs of their own play, implying that the well-being benefits they gain may be reflected in the financial resources they willingly invest in the sport. What is it about golf that makes it so valuable to its players that they choose to invest both time and money into their hobby? As golf's popularity continues to grow globally, particularly among adults who account for a significant share of healthcare costs (6) and recreational spending, understanding golf's broader social and economic significance becomes increasingly important. A key rationale for applying SROI methodology to golf is that participants typically finance their own participation, yet simultaneously generate broader societal benefits that are not captured by traditional economic measures (7, 27).

The Finnish Golf Union is the largest sport union in Finland in terms of registered membership (28), with 161,635 members (29), representing about 3% of the total population. In 2022, there were 154,415 members (30), indicating a 4.7% growth over 2022–2025. Golfers comprised 2.8% of the population in 2022 and remained at the same level in 2025 (31, 32). Based on Finnish Golf Union data, the growth in membership and played rounds reflects golf's strong appeal (28, 29). The peak volume of rounds occurred in 2020 during the COVID-19 pandemic, when golfers played 4,127,226 rounds, 39% more than in 2019. In comparison, 3,388,833 rounds were played in 2022. The mean age of golfers in Finland is 48 years (30), with players, particularly men, evenly distributed across age groups from 20 to 70 years.

This study aimed to estimate the social value of golf in Finland by analysing the social return on investment (SROI) of golf in 2021 across two dimensions: (1) the financial flows generated by Finnish golf players’ spending across various economic sectors, and (2) the wellbeing and physical fitness promoted by golf participation and their broader effects on societal sectors such as healthcare services. The findings aim to provide evidence-based insights for policymakers, sports organisations, and other stakeholders on the societal and economic contribution of golf.

## Methods

### Research questions

This study analyses the social significance of golf in Finland. There is currently no consensus on which social outcomes should be measured and how they should be evaluated (2), so in this study context “social significance” refers to two main aspects: (1) the wellbeing, health, and physical fitness promoted by golf, and their effects on different sectors of society such as healthcare services, and (2) the financial contributions from golf players to various sectors of society. A comprehensive analysis of the physical activity promoted by golf has been published in a previous article (22). To assess the wellbeing, health, and physical fitness promoted by golf and their effects on societal sectors such as healthcare services, this study uses Kolu et al. (10) as reference data.

This article addresses the following research questions:

1. How much money do Finnish golf players spend on golf, and how does this spending flow into various economic sectors?
2. What is the Social Return on Investment (SROI) ratio of golf when comparing the funds invested to the financial inflows and broader economic impacts generated by golf?

### Data collection

The main data for the study were collected through an online survey in May 2022. The questionnaire was published in Finnish and Swedish and was tested by four players and/or experts who did not participate in its design. Invitations to participate in the survey were sent to 10,000 members of the Finnish Golf Union, followed by a second invitation to an additional 4,000 members, which was slightly more targeted at sub-groups of the player population that were underrepresented among the initial respondents. In addition to the survey data, the analysis utilized existing research, financial statements, and national statistics. All monetary values are derived from monetary transactions or costs and savings reasonably assessed from these data sources, as summarized in Table 1.

**Table 1:** Data sources used in the analysis.

Data	Source
Golf spending, physical activity levels, motivations, joy of playing	Online survey (2022)
Societal cost of physical inactivity	Kolu et al. (10)
Golf business financial data	Financial statements of 10 golf courses, interview of experts
Equipment imports/exports	Statistics Finland (31)
Economic multiplier	Leontief coefficient, Statistics Finland (31)

### Questionnaire survey

The questions were divided into three main topics: (1) physical activity, (2) spending on golf, and (3) the impact of golf on personal wellbeing and the motivations for playing golf. Additionally, the questionnaire gathered background information, including age, gender, household size, educational attainment, employment status, home province, and household gross income.

The first set of questions collected information about the role of golf as a component of weekly and yearly physical activities, along with data that enabled the assessment of the role of golf in various phases of the lifespan. Respondents were asked to assess the total amount of all their weekly physical activities throughout the year and to report other physical activity providing hobbies besides golf. Kolu et al. (10) serves as reference data for comparing the physical activity levels of golf players to average activity levels among the Finnish population. Sedentary lifestyle impacts were excluded from the analysis for two reasons: (1) respondents were not asked about their sedentary time duration, and (2) Kolu et al. (10) did not quantify the overlap between physical inactivity and sedentary behaviour. The wellbeing, health, and physical activity promoted by golf is published in a previous article (22).

The second set of questions focused on spending on golf. The questions encompassed the following items: 1) costs directly associated with playing golf and maintaining a golf club membership, such as shareholder payments, game fees, golf equipment rentals, and similar expenditures; 2) expenditures linked to practice sessions, participation in golf courses, coaching, and related activities; 3) expenditures on lunches, snacks, and other refreshments within the golf club; 4) golf-related travel within Finland; 5) purchases of golf equipment both in Finland and abroad; and 6) participation fees for golf tournaments. Respondents were also asked about their golf-related travel within Finland and abroad. Recognizing that respondents might not possess exact knowledge of their spending amounts, they were asked to select a predetermined spending range. The survey used a logarithmic scale for most spending-related questions, featuring six alternative categories: “less than 200 euros”, “200–400 €”, “400–800 €”, “800–1,600 €”, “1,600–3,200 €”, and “more than 3,200 €”. The logarithmic scale was chosen because estimation errors are more substantial at higher spending levels. For example, someone spending 100 euros annually might estimate between 50 and 150 €, whereas at 1,000 € annual spending, the estimation range might extend from 500 to 1,500 €.

### Social return on investment ratio calculation

The SROI ratio is calculated by dividing the assessed value of benefits by the value of investment (Table 2). Previous SROI studies in physical activity and sport have most commonly measured outcomes related to health, subjective wellbeing, social capital, education, and crime reduction (1, 9). The present study selected outcomes based on their quantifiability and relevance to the Finnish context, focusing on healthcare cost savings, institutional care costs, workforce productivity, and tax revenues, consistent with the cost framework established by Kolu et al. (10). If the ratio exceeds one, the investment can be considered positive for society. In cases where utilization does not involve direct monetary transactions, alternative valuation methods are employed, such as determining how much the beneficiary would be willing to pay for an alternative product or service (33–35). In this analysis, all monetary values are derived from monetary transactions or costs and savings reasonably assessed from existing data, including this survey, other research, statistics, and expert assessments.

**Table 2:** Investment and benefit components of the SROI ratio.

Investment or Benefits	Component
Investment	Players’ total expenditure on golf
Benefits	Subjective joy and wellbeing provided by golf to its players
Benefits	Financial flows to Finland from purchases of golf equipment and gear
Benefits	Salaries, fees, other expenses, and investments paid by golf courses
Benefits	Financial flows to domestic tourism-related services from golf tourism within Finland
Benefits	Savings and additional revenues to society generated by golf

The SROI calculation comprises two main components. First, total expenditure on golf is estimated. Second, an assessment of the financial flows generated for society is presented. Investments in golf are assumed to equal players’ expenditures, including fees related to playing, recreational services offered by golf courses, purchases of golf equipment, and domestic golf travel.

The benefits generated by golf for different stakeholders include the following: the subjective joy and wellbeing experienced by players, estimated on the basis of players’ expenditure on golf; the financial flows to Finland generated by purchases of golf equipment and gear; salaries, fees, other operating expenses, and investments paid by golf courses; the financial flows to domestic tourism-related services generated by golf tourism within Finland; and the savings and additional revenues to society generated by golf.

A critical question is whether spending on golf truly measures the level of joy and wellbeing. Not directly; however, it indicates how much players are willing to pay in order to obtain the personal benefits associated with playing. Ala-Vähälä et al. (22) reported, based on the same dataset as in this article, that the main motivations for playing golf were joy, wellbeing, and the opportunity to spend time with friends and loved ones. In other words, the amount of spending may not directly indicate the level of joy and wellbeing, but it does indicate that players are committed to the hobby and willing to pay for it, because playing brings them joy and well-being.

In some cases, individuals who have paid golf club membership fees and purchased golf equipment may be unable to play because of illness, lack of motivation, or reasons related to advanced age. In such cases, the money spent does not yield the expected benefits. This issue will be considered later when the results of the SROI assessment are presented.

Based on these components, the SROI ratio of golf is calculated as

SROI = Total societal benefits from golf/Total spending on golf

To illustrate, if the joy derived from playing golf equals the amount spent on it and no money flows abroad, the total social value would be twice the spending, yielding an SROI of 2 × spending/spending = 2. In reality, three key factors modify this theoretical ratio. First, a significant share of golf equipment is imported, reducing the ratio by diverting funds abroad. Second, golf generates indirect economic benefits through improved health and fitness, which reduce healthcare costs and increase the SROI. Third, spending on golf creates multiplier effects as golf clubs invest, purchase goods and services, and pay salaries that support local and national businesses, further increasing the SROI.

### Calculation of societal savings from physical activity

Based on the same dataset used in this article, Ala-Vähälä et al. (22) reported that Finnish golfers demonstrated substantially higher physical activity levels than the general population, with 89% engaging in at least four hours of weekly physical activity throughout the year and 59% participating in vigorous-intensity activities for more than two hours per week. Nearly 90% of respondents engaged in at least one additional type of physical activity alongside golf throughout the year, and 70% reported one or two such additional activities. Of all respondents, 18% considered golf their secondary activity, reporting higher levels of participation in other sports or exercise (22).

First, projected societal costs associated with low levels of physical activity were determined by gender and age group, assuming that physical inactivity levels among golf players were equivalent to those of the general population (Sum A). Second, a similar approach was used to estimate the costs of low physical activity levels among golf players based on the actual proportion of inactive individuals from the survey data (Sum B). The estimated societal savings and increased income were obtained by subtracting Sum B from Sum A.

Societal savings = Sum A−Sum B

The analysis also incorporated the Leontief coefficient for sports activities and amusement and recreation services (1.7) from Statistics Finland (43). This coefficient represents the ratio of total economic output to direct economic inputs, capturing multiplicative effects throughout the value chain. The multiplier-adjusted estimate is presented as a supplementary economic-impact scenario that illustrates the potential broader economic ripple effects beyond direct expenditures, rather than as a definitive measure of the true societal return on investment.

### Statistical analysis

All figures originally reported in USD or GBP have been converted to EUR using January 2026 average exchange rates (1 USD = 0.8519 EUR; 1 GBP = 1.1547 EUR) (37). Descriptive statistics of study participants’ characteristics and golf-related data were analyzed using IBM SPSS Statistics version 27.0 (Armonk, NY: IBM Corp). Means and standard deviations were calculated for continuous variables, while frequencies and percentages were calculated for categorical variables. The Kolmogorov–Smirnov test and visual histogram inspection indicated non-normally distributed data; therefore, Mann–Whitney U test was used to determine gender differences in continuous parameters. Age group differences in categorical variables were assessed using chi-square tests. For economic and SROI calculations, all analyses were performed using Microsoft Excel (version 2021). The significance level was set to *p* < 0.05, and confidence intervals were 95%.

## Results

### Characteristics of study participants

The survey received 1,052 responses, resulting in a response rate of 8%. The respondents included 64% males (mean age 54 years, SD 16 years) and 35% females (mean age 56 years, SD 15 years), with an overall average age of 55 years (SD 16 years) and an age range of 18 to 87 years. Female respondents were slightly older than men (*p* = 0.044). Respondents were categorised into six age groups (18–29, 30–39, 40–49, 50–59, 60–69, >70 years), with a statistically significant difference observed in response rates between groups (*p* = 0.042). Compared to the base population of Finnish Golf Union members (38), the age group of 60–69 years was over-represented in men and women's, while younger age groups were under-represented, particularly 18–29 years. The age groups of 40–49 and 50–59 years were broadly representative of the Golf Union membership in men and women's.

The mean handicap was 22 (SD 11) for men and 28 (SD 9) for women, both lower than the Golf Union membership averages of 25 and 35 respectively (*p* < 0.001). The observed difference was larger among women, which may suggest that female respondents were more committed to the sport than the average female Golf Union member. For 75% of respondents, the active play season typically lasted 4–7 months, with the most common duration being 5 months (*n* = 246, 23%) and the second most common 6 months (*n* = 213, 20%). During their active play season, over 70% of respondents played golf at least once a week. Across nearly all age groups, approximately 90% or more of respondents expected to maintain or increase their participation level over the next five to ten years.

Overall, 89% of respondents reported engaging in some form of physical activity for at least four hours per week throughout the year, and 59% participated in vigorous-intensity activities for more than two hours per week. Among both men and women, the proportion of respondents meeting national physical activity guidelines was higher for golf players than for the general population.

Respondents were also asked about the benefits and enjoyment they experienced from playing golf. The highest scores were given to direct benefits such as joy and the opportunity to spend time with family, friends, or other close people. Indirect benefits, including improvements in health and fitness, opportunities to make new friends, and advantages in practicing other sports or physical activities, were also positively reported, although not as highly as the direct benefits.

### How much do players spend on golf?

The estimated total golf consumption in Finland for the year 2021 is presented in Table 3. The most significant expense category consisted of direct costs associated with playing and maintaining a golf club membership. Typically, individual players spent between €800–1,600 on membership fees, game fees, and similar items (44% of respondents), with the second most common response being €400–800 (25% of respondents). Golf equipment purchases represented the second largest spending category, with 37% of respondents spending €200–400 annually, while another significant proportion spent less than €200. International equipment purchases were relatively minor, with 74% of respondents making no purchases from abroad in 2021 and 15% spending less than €200 on international purchases.

**Table 3:** The estimated total golf consumption in Finland for the year 2021.

Category of spending	M €
Shareholder fees, other fees related to playing, practicing, rent of equipment	150
Purchases on golf equipment in Finland	59
Golf travel in Finland	50
Spending in club restaurant, other refreshment at the golf club	33
Purchase abroad (foreign online shops included)	16
Coaching, golf courses and other instruction	15
Participation fees on golf tournaments	9
Total	332

Expenses for practicing, coaching, and refreshments were considerably smaller. More than half (53%) of respondents did not spend anything on practicing and coaching, while 28% spent less than €200. Regarding refreshments, respondents reported an average spending of approximately €11 per club visit, which totaled approximately €33 million in 2021 when considered across all players’ frequency of play.

Golf-related travel constituted another spending category. Regarding golf tourism abroad, nearly half of respondents (46%) did not travel for golf in 2021, while 19% reported one trip and 14% reported two trips. For domestic golf travel within Finland, 74% of respondents provided spending information: 30% spent less than €200, 28% spent €200–400, and 25% spent approximately €400–800.

Tournament participation also generated expenses, although these were modest. Approximately half of respondents had participated in one or more tournaments, with highest participation among respondents in their 40s and 50s. Among those who participated, 80% paid less than €200 in tournament fees.

Total consumption was calculated by selecting the midpoint of each spending category, except for the highest category, which was assigned a value of €3,200. Using survey data, estimates were made to determine the proportion of golf players in each spending category, stratified by age group and gender. The analysis revealed that the most significant expenditures were membership and game fees, followed by golf equipment purchases and domestic golf travel.

### How does the money spent on golf further flow through society?

The flow of money from golf to society is analyzed from two perspectives. First, it examines the primary expenses of golf courses, including allocations to salaries, operational expenses, and investments. Second, it investigates how much money from golf equipment purchases remains in Finland versus flows abroad, examining the impact on the trade balance. According to the survey analysis above, golf players spent approximately €150 million on membership fees, game fees, and related expenses. While golf courses may have other income sources, the following analysis of the financial contributions from golf courses to the Finnish economy assumes this figure as a rough estimate of their total turnover.

Comprehensive statistics regarding the cost structure of Finnish golf courses are unavailable. Therefore, an analysis of financial statements from ten golf courses was conducted. Within this group, salary expenses averaged 38%, accounting for approximately €57 million of the total golf course turnover. Other operational expenses necessary to run the business accounted for approximately 35% of turnover, much of which likely benefited local suppliers. The remainder comprised depreciation, write-downs, and profits or losses for the financial year. According to expert interviews, constructing a golf course in Finland costs approximately €3–5 million (2021 prices), suggesting that Finnish golf courses have a total value of at least €500 million. Assuming a 25-year construction and renovation cycle, annual investment costs would be approximately €20 million, roughly equivalent to the depreciation and write-down share (14% of approximated turnover).

Previously, it was noted that golf enthusiasts spent approximately €76 million on golf equipment, comprising purchases in Finland and abroad. According to Finnish Customs statistics, the net import value was approximately €17 million in 2021. Of the total consumption, at most approximately €43 million remained in Finland, while at least approximately €33 million went abroad. The outflow abroad comprised three components: imports (€17 million), purchases from foreign online stores (€11 million), and purchases made abroad (€5 million). The import share may be larger, as these statistics did not account for golf clothing and footwear.

### The indirect economic impacts of golf

Beyond direct spending, golf generates broader societal savings through the higher physical activity levels of its participants. The assessment of the total value of societal savings and added incomes due to the smaller share of inactivity among golf enthusiasts is presented in Table 4.

**Table 4:** Assessment on the total value of societal savings and added incomes due to smaller share of inactivity among golf enthusiasts.

Category	The age group that was included in the assessment	Savings or increased tax-revenues from active lifestyle, M€
Increase of tax revenue	19–64 years	46.0
Institutional care for the elderly	65–84 years	11.0
Premature mortality	19–64 years	7.5
Disability pensions	19–64 years	8.1
Use of health care services	19– years and over	5.2
Medical expenses	19– years and over	1.2
Long-term sick leaves	19–64	1.1
Unemployment benefits	19–64 years	0.5
Short-term absences from work	19–64 years	0.3
Total		80,9

The total value of societal savings and added incomes is approximately €80 million. The largest single category, increased tax revenue (€46.0 million), reflects the contribution of golf to workforce productivity, while reduced institutional care costs for older adults (€11.0 million) and disability pensions (€8.1 million) represent the most significant healthcare-related savings.

### The SROI analysis of golf

The main expenses of playing golf in 2021 consisted of shareholder fees and other fees related to playing golf (approximately €150 million), purchases of golf equipment in Finland (€59 million), golf travel in Finland (€50 million), and spending in club restaurants and other refreshments at golf clubs (€33 million). The total value of consumption was €332 million, which was rounded to €330 million for this SROI calculation. In the long run, golf clubs utilize all collected funds in diverse ways. However, it was feasible to trace only approximately €250 million of the total consumption of €330 million. After subtracting the value of imported golf equipment, approximately €220 million remained to contribute to Finnish society, as illustrated in Table 5.

**Table 5:** Money flows from golf to Finnish economy (M€).

Category	The main beneficiaries	M€
Wages and salaries	Employees (+ salary related payments to state)	57
Other consumption related to running the golf courses	Local subcontractors and suppliers	50
Golf related travel in Finland	Hotels, restaurants etc.	50
Purchase of golf equipment	Assessment of the net value that stays in Finland	43
Investments	Builders and suppliers of investment goods	20
Total		220

Beyond these direct monetary flows, golf also generates indirect savings and revenues across various sectors of society, estimated at approximately €80 million, as detailed in the Indirect Economic Impacts section. The overall benefits encompass the value of joy and wellbeing for players (€330 million), money flows to the Finnish economy (€220 million), and savings and added revenues to society (€80 million), resulting in a total of €630 million. The assessed value of investments in golf amounted to €330 million. From these figures, the SROI ratio in Finland is approximately €630 million divided by €330 million, yielding a ratio of 1.9.

The actual ratio is likely to be greater for two reasons. First, the money flows to the Finnish economy encompass only those items that could be assessed. As mentioned earlier, the impact is likely larger because golf clubs allocate all income collected from members and other sources over time. Second, when multipliers are included, economic impact may increase through local economies, as golf clubs increase demand for goods and services, which in turn stimulates economic activity. According to Statistics Finland, the Leontief coefficient for sports activities and amusement and recreation services is 1.7. As a supplementary economic-impact scenario, applying this multiplier would increase the monetary estimate from €220 million to approximately €370 million, which would raise the SROI ratio from 1.9 to 2.4.

What is the role of joy and wellbeing in this calculation? In other words, how does the SROI value change if players no longer experience joy and wellbeing from playing golf? To assess this, a sensitivity analysis was conducted using three scenarios: (1) all players experience joy and wellbeing, (2) 50% of players experience joy and wellbeing, and (3) none of the players experience joy and wellbeing.

It was assumed that players would continue to pay membership fees but would reduce other golf-related purchases proportionally. The estimated health-related savings were assumed to decrease proportionally. The golf-clubs would continue their activities, paying wages and making purchases. Tables 6, 7 present the results without the Leontief multiplier (Table 6) and with the Leontief multiplier (Table 7).

**Table 6:** Sensitivity analysis of SROI-value without Leontief-multiplier.

Row	Spending, assessed value or economic benefit	Full value	50% value	0% value
1	Shareholder fees	150	150	150
2	Joy/shareholder fees	150	75	0
3	Joy/ purchases in Finland	59	29,5	0
4	Joy/ golf travel in Finland	50	25	0
5	Joy/ spending in restaurants etc.	33	16,5	0
6	Joy/ purchases abroad	16	8	0
7	Joy/ coaching, golf courses etc.	15	7,5	0
8	Joy/ participation fees	9	4,5	0
9	Total assessed value of joy and wellbeing (Rows 2–8)	332	166	0
10	Health related savings	80,9	40,45	0
11	Vages	57	57	57
12	Other consumption (golf clubs)	50	50	50
13	Travel	50	25	0
14	Purchase of golf equipement	43	21,5	0
15	Investments (golf clubs)	20	20	20
16	The total value of benefits (Rows 9–15)	632,9	379,95	127
17	The total value of inputs (Rows 1, 3–8)	332	241	150
18	SROI-value (Row 16/row 17)	1,9	1,6	0,8

**Table 7:** Sensitivity analysis of SROI-value with Leontief-multiplier.

Row	Spending, assessed value or economic benefit	Full value	50% value	0% value
1	Shareholder fees	150	150	150
2	Joy/shareholder fees	150	75	0
3	Joy/ purchases in Finland	59	29,5	0
4	Joy/ golf travel in Finland	50	25	0
5	Joy/ spending in restaurants etc.	33	16,5	0
6	Joy/ purchases abroad	16	8	0
7	Joy/ coaching, golf courses etc.	15	7,5	0
8	Joy/ Participation fees	9	4,5	0
9	Total assessed value of joy and wellbeing (Rows 2–8)	332	166	0
10	Health related savings	80,9	40,45	0
11	Vages	96,9	96,9	96,9
12	Other consumption (golf clubs)	85	85	85
13	Travel	85	42,5	0
14	Purchase of golf equipment	73,1	36,55	0
15	Investments (golf clubs)	34	34	34
16	The total value of benefits (Rows 9–15)	786,9	501,4	215,9
17	The total value of inputs (Rows 1, 3–8)	332	241	150
18	SROI-value (Row 16/row 17)	2,4	2,1	1,4

The sensitivity analysis indicates that under the 50% scenario, the SROI value would be 1.6 (2,1) when the Leontief multiplier is included). Under the zero-joy-and-wellbeing scenario, the SROI value would decrease to 0.8 (1,4 when the Leontief multiplier is included).

## Discussion

This study provides one of the first comprehensive estimates of golf's societal and economic significance in Finland, by employing the Social Return on Investment (SROI) methodology to quantify outcomes that cannot be directly measured in monetary terms (7). The analysis addressed two primary research questions: (1) How much money do Finnish golf players spend on golf and how does this spending flow through various economic sectors? and (2) What is the SROI ratio of golf when comparing invested funds to financial inflows and broader economic impacts? Previous research, particularly the Social value of golf in the UK final report (27), represents one of the first comprehensive SROI analyses of golf participation. In the field of sports, SROI analyses have been conducted by the Football Association of Finland (39) and the Finnish Floorball Federation (40). However, the sources and methods differ substantially between studies. As Davies et al. (2) emphasize, there is currently no consensus on which social outcomes should be measured and how they should be evaluated, and SROI calculations remain context-specific rather than standardized. Despite these methodological limitations, SROI remains a useful tool for evaluating and communicating the societal significance of investments (34).

### The SROI analysis of golf

Golf enthusiasts primarily finance their hobby themselves, and the value golf generates for golfers is assumed to equal the amount they spend. The willingness-to-pay principle assumes that if a golfer spends €3,898 annually (€330 million/161,635 members), that spending reveals the personal value they place on the activity. In this analysis, value refers to the monetary equivalent of benefits reflecting willingness-to-pay, while benefits denote measurable outcomes such as improved health, reduced costs, and increased productivity. In cases where utilization does not involve direct monetary transactions, alternative valuation methods are employed, such as determining how much the beneficiary would be willing to pay for an alternative product or service (33–35). Survey respondents ranked joy of playing and time with friends/loved ones as the highest perceived benefits, while professional networking ranked lowest, indicating that golfers spend primarily for intrinsically rewarding experiences such as enjoyment, social connection, improved psychological and physical condition, skill improvement, and mastery (14) rather than instrumental outcomes. However, the SROI analysis extends beyond personal value to demonstrate that society receives substantial additional benefits beyond golfer enjoyment. Some individuals may own golf club shares but be unable to play due to age or health issues, making actual value slightly lower than total spending, though this factor is expected to be insignificant.

The SROI analysis comprised three components, namely golfer joy and wellbeing (€330 million), monetary flows to the Finnish economy (€220 million, as detailed in the Economic Contributions section), and societal savings and additional revenues (€80 million, as detailed in the Health and Social Benefits section), resulting in an estimate totaling €630 million. This breakdown highlights an important finding that golfers do not fully internalize the societal value they generate. They spend €330 million for personal benefits worth €330 million, but their participation simultaneously generates €300 million in external societal benefits accruing to society rather than golfers themselves. In economic terms, golf generates substantial positive externalities. The SROI ratio was calculated as €630 million divided by €330 million, yielding a ratio of 1.9. When multiplier effects are incorporated using the Leontief coefficient of 1.7 as a supplementary economic-impact scenario, the estimated economic impact increases from €220 million to approximately €370 million, expanding total benefits to approximately €770 million and raising the SROI ratio to approximately 2.4.

**Figure 1:**
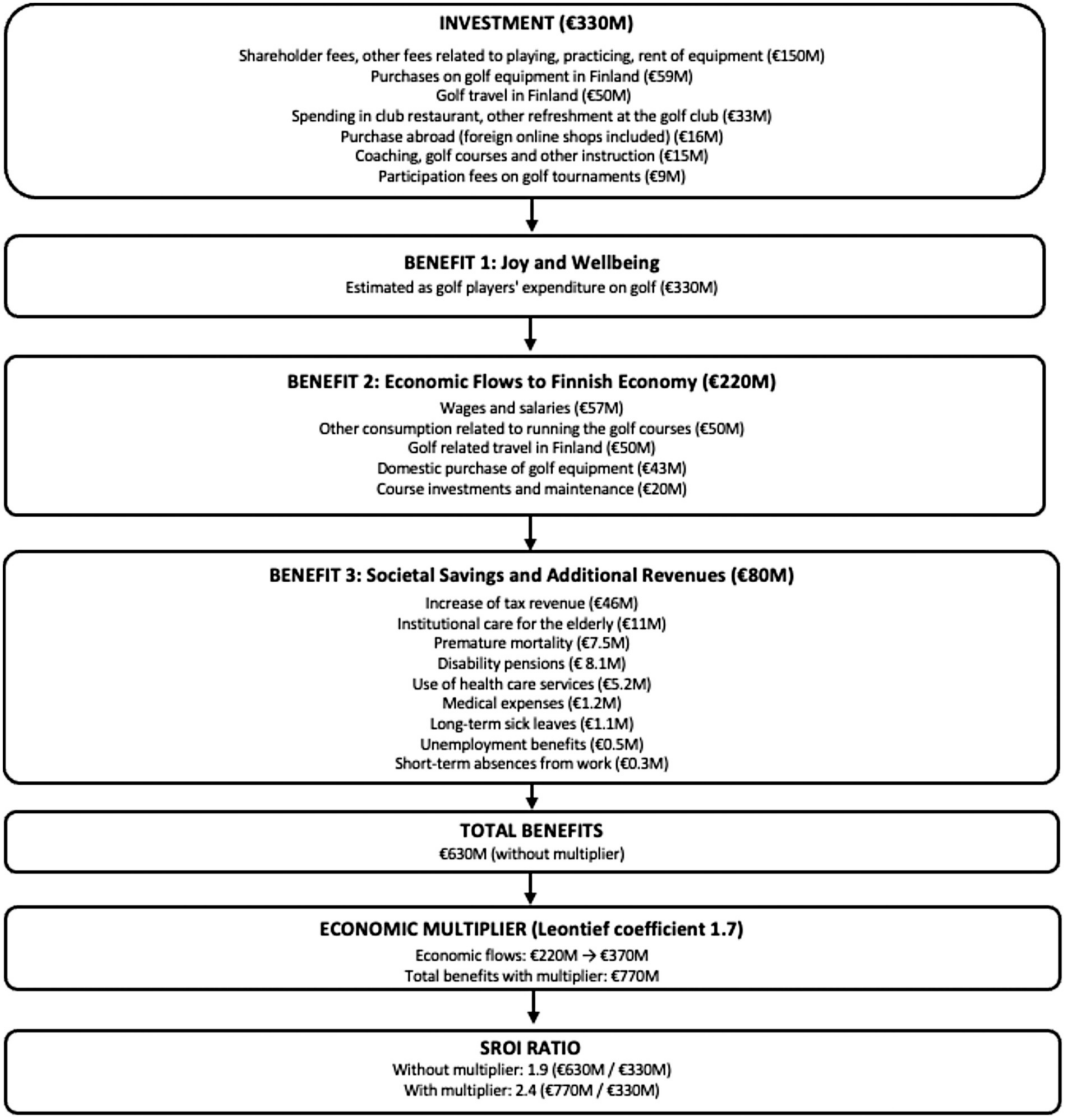
Flowchart of the SROI analysis.

Finland's SROI estimate of 1.9 to 2.4 aligns with comparable sports and physical activity spending 1.92 to 3.28 (27, 33). The UK golf SROI analysis (27) reported higher absolute value (€1.20 billion) but employed different methodologies emphasizing subjective wellbeing and community development rather than healthcare and tax quantification, while Finland's explicit economic accounting yields higher per-capita value (€3,898 versus approximately €1,200). However, the lack of standardized SROI methodology (2) means these figures cannot be directly compared. Rather, they indicate that golf generates substantial societal value relative to golfer spending. The results support public policies promoting recreational golf access, particularly among middle-aged and older adults (1, 2). The overall structure of the SROI analysis is illustrated in Figure 1.

Some limitations regarding sample representativeness should be acknowledged when interpreting the results. The survey response rate of 8% is relatively low, and the average handicap of participants was significantly better than the national average, suggesting that the sample likely overrepresents more committed and experienced golfers (41). This is relevant from an economic perspective, as Hallmann & Wicker (42) found that handicap, time spent playing, and income are significant predictors of golf-related expenditure, with more skilled and active golfers spending considerably more than recreational players. Consequently, the per capita spending estimates and the resulting SROI ratio may be slightly overestimated compared to what would be observed in a more representative sample, and future studies should aim to capture a broader range of golfer profiles.

### Economic contributions

In 2021, Finnish golfers spent approximately €330 million on golf. This spending circulated broadly through the Finnish economy, supporting wages and salaries (€57 million, representing 38% of golf course turnover), local suppliers and operational expenses (€50 million), domestic golf equipment retail (€43 million), golf tourism including hotels and restaurants (€50 million), and course investments and maintenance (€20 million). These results show that golf spending reaches several different economic sectors, consistent with international evidence showing US golf generating €193 billion in total economic activity in 2022 (15) and European golf impact estimated at €15 billion (25).

The total turnover of the golf business was estimated at approximately €150 million based on respondents’ reported spending (28). A review of financial statements from ten golf companies confirms that salaries and wages accounted for approximately 38% of turnover, while operational spending represented approximately 35%, much of which likely benefited local suppliers. Of approximately €76 million in total equipment spending, at least €33 million flowed abroad, while at most €43 million remained in Finland (36), reflecting the import-dependent nature of golf manufacturing. Annual course investment costs of approximately €20 million appear consistent with observed depreciation levels, though whether these investments adequately meet long-term renewal needs warrants further attention.

Although 2021 spending patterns may reflect COVID-19 influences on golf tourism, the Finnish Golf Union’s reported 4.7% membership growth from 2022 to 2025 suggests the pandemic activated a durable increase in participation rather than simply shifting future demand forward. As outdoor settings offered safe social distancing and golf continued to fulfill fundamental motivational needs (11), these participation trends support the expectation of sustained economic engagement with golf going forward.

### Health and social benefits

Finnish golfers demonstrated substantially higher physical activity levels than the general population, with 89% engaging in at least four hours of weekly physical activity throughout the year and 59% participating in vigorous-intensity activities for more than two hours per week (22). The respondent group had a mean age of 55 years with the largest concentration in the 50 to 69 age group, which is noteworthy given that prior research has identified middle-aged and older adults as representing the largest potential age group for reducing healthcare costs through increased physical activity (6). Golf appears particularly well-suited to this demographic, as it combines moderate-intensity aerobic exercise, muscle engagement, cognitive stimulation, and social interaction (13, 16–18), supporting disease prevention, improved psychological and cognitive function, and prosocial behavior (1, 3, 19, 20).

These higher activity levels have measurable implications for societal costs, via our model's assumption that regular physical activity is directly attributable to health care cost savings. Physical inactivity represents a major modifiable risk factor for noncommunicable diseases and mental health conditions (4), and is associated with substantial societal costs across healthcare, long-term care, lost work productivity, and social security expenditures (5). Prior Finnish research estimated that insufficient physical activity costs Finnish society approximately €3.2 billion annually (10). Using this framework, the present analysis estimated that golf participation contributed approximately €80.9 million in annual societal savings and additional tax revenues. The savings included reductions in healthcare service utilization and medical expenses (€5.2 to 6.4 million), institutional care costs for older adults aged 65 to 84 (€11.0 million), work absences and disability pensions (€9.5 million), as well as increased tax revenues from improved workforce participation (€46.0 million) and reduced unemployment benefit expenditures (€0.5 million). The largest single category, increased tax revenue at €46.0 million, reflects golf's contribution to workforce productivity beyond direct healthcare savings.

This potential reduction in institutional care costs for older adults is particularly relevant to broader policy discussions. Research has suggested that prevention of physical inactivity-related diseases might paradoxically increase societal costs if people live longer and require care for extended periods (5). However, the present data suggest this is offset by older golfers maintaining better functional capacity and requiring less care despite longer lifespans. This leads us to assume that golf participation may be economically beneficial to society even when accounting for extended lifespan. Golf could play a valuable role as a physical activity option particularly available to and attractive to people in middle and later stages of life, making it potentially important for maintaining health and productivity in an aging population (1, 6).

It is important to recognize that different types of benefits accrue to different sectors of society. Tax revenue increases and changes in healthcare and elder care expenditures primarily affect the public sector, while reductions in work absences represent broader productivity gains (10). However, the relationship between physical activity and economic outcomes involves significant complexities and uncertainties. Benefits of today's golf participation will primarily accrue in the future, whereas measured costs reflect decisions made in earlier years. Uncertainties remain regarding how past activity patterns influence current health, how current levels translate into future benefits, and how healthcare systems will evolve (4, 5).

### Participation motivation and long-term engagement

A striking feature of golf participation in this study was its long-term sustainability. Over 75% of respondents maintained active play seasons lasting 4 to 7 months, with more than 70% playing at least once weekly during their season, and approximately 90% across nearly all age groups indicated they planned to maintain or increase their involvement over the next 5 to 10 years. This sustained engagement pattern is important for understanding golf's potential health impact, since cumulative benefits of physical activity typically require consistent participation over time (6).

The joy of playing and spending time with friends and family were reported as the most important benefits of golf, while health and fitness improvements, opportunities to make new friends, and the way golf influenced participation in other physical activities were rated as secondary but still meaningful. In contrast, professional networking or career advancement was rated as least important. These responses suggest that intrinsic enjoyment and social connection are the primary drivers of participation (14), which may partly explain the high rates of sustained participation observed in this population (1). Intrinsic motivation, the tendency to engage in activity for its own sake rather than external rewards, is known to be particularly important for long-term physical activity adherence (3).

### Economic multiplier effects

Economic multiplier effects may substantially expand the measured direct impact of golf spending. As a supplementary economic-impact scenario, applying Finland's Leontief coefficient of 1.7 for sports and recreation services suggests that the direct economic flows of €220 million could expand to approximately €370 million in total economic impact, as golf clubs purchase goods and services from suppliers who in turn generate further demand throughout the economy. Beyond direct spending, golf also generates indirect economic impacts through enhanced physical activity and associated health outcomes, as golfers are generally more active than the general population (1, 3), with measurable consequences for healthcare costs and workforce productivity (5, 10). Combined with the 56% of equipment spending retained in Finland, these findings indicate long-term, sustained economic engagement with the sport.

These estimates carry several important caveats. First, the physical activity threshold applied (4 h per week) exceeded standard Finnish guidelines (2.5 h), mitigating the risk of overestimated activity levels. Second, wage assumptions were based on national averages, though golfers typically earn above-average incomes, suggesting that calculated tax revenue gains may underestimate the economic benefit. Third, age groupings do not completely align with those in the original cost studies (10), introducing minor uncertainty. Fourth, changes in income tax rates would alter projected revenue gains independent of activity levels. Fifth, even if most golfers meet weekly activity recommendations, some may lead otherwise sedentary lifestyles, potentially diminishing the health benefits of their golf participation.

### Limits of the study

This study contains several limitations. Spending estimates derive from self-reported survey data, which may introduce recall and estimation bias, particularly for non-routine expenditures such as golf travel. The survey response rate of 8% is relatively low and may limit the representativeness of the sample. The significantly lower average handicap of participants compared to the national average suggests that more committed and experienced golfers were overrepresented, and they may be more prepared to spend money than higher handicap players, which may have resulted in slightly higher spending estimates and, consequently, an upward bias in the SROI ratio. The attribution of physical activity benefits to golf is limited, as golfers typically engage in multiple physical activities. Temporal asymmetry exists in the analysis: present-day costs of historical inactivity are compared against present costs of current activity maintenance, whereas benefits from current golf participation will materialize over time. The SROI methodology applied is non-standardized and context-dependent, limiting direct comparability with other studies. Environmental externalities, particularly carbon emissions from golf-related travel, were not monetized and thus remain unincorporated. The model assumes that all benefits are accrued entirely from golf related physical activity and this excludes the potential for any other types of physical activity to contribute to benefits. The analysis employs a 4-hour weekly physical activity threshold that exceeds standard Finnish recommendations (2.5 h) and uses national average wages despite evidence that golfers earn above-mean incomes. These assumptions suggest the estimated societal benefits may be conservative estimates rather than overestimates of golf's impact.

## Conclusion

This study demonstrates that golf could generate substantial social return on investment in Finland, with an SROI ratio of 1.9 (€630 million in benefits from €330 million in spending), increasing to 2.4 when economic multiplier effects are incorporated. Finnish golfers, characterized by higher physical activity levels than the general population and sustained long-term participation, could generate approximately €80.9 million in annual societal savings through reduced healthcare and care costs, while golf spending circulates approximately €220 million through the Finnish economy via employment, services, and investments. These findings reveal that golf may create substantial positive externalities approximately €300 million in societal benefits beyond individual golfer benefits supporting policy initiatives promoting recreational golf access, particularly among middle-aged and older adults. The model does have methodological limitations inherent to non-standardized SROI assessments and conservative assumptions regarding activity thresholds and wage-based calculations, however the analysis provides useful empirical evidence that golf participation could contribute significantly to population health, economic activity, and societal wellbeing. We ask other golf nations across the globe to examine their data sources and epidemiological research to also estimate golf's role as a valuable potential public health intervention for reducing the societal burden of physical inactivity.

## Data Availability

All data analyzed during this study are included in this published article.
